# Exploring the perspectives of allied health practitioners toward the use of journal clubs as a medium for promoting evidence-based practice: a qualitative study

**DOI:** 10.1186/1472-6920-11-66

**Published:** 2011-09-23

**Authors:** Lucylynn M Lizarondo, Karen Grimmer-Somers, Saravana Kumar

**Affiliations:** 1 International Centre for Allied Health Evidence, University of South Australia, North Terrace, Adelaide, 5000, Australia

## Abstract

**Background:**

Research evidence suggests that journal clubs (JCs) are one approach which can be used to bridge the gap between research and clinical practice. However, there are issues which potentially threaten their viability such as on-going participation or compliance with attendance, which require further exploration. The objectives of this study are: to explore the views and perspectives of allied health practitioners (AHPs) regarding the use of any type of JC in promoting evidence-based practice (EBP); to identify ways in which an innovative model of JC developed by the International Centre for Allied Health Evidence (*i*CAHE) might be refined.

**Methods:**

A qualitative descriptive study utilising focus group interviews with various groups of AHP was undertaken-- those who have been exposed to the *i*CAHE JC model and those who have no experience of the *i*CAHE model (although they may have had exposure to other forms of JC). Maximum variation sampling was used to recruit participants for the study. Transcripts of focus groups were coded and distilled into content-related categories.

**Results:**

Six focus groups with 39 AHPs were facilitated. Allied health practitioners perspectives' on JCs were classified in five broad categories: utility and benefits of a JC, elements of an effective and sustainable JC, barriers to participation, incentives for participation, and opportunities for improvement in the current *i*CAHE JC model. Overall, JCs were seen as a forum for reflective practice and keeping up-to-date with research evidence, and a venue for learning the processes involved in critical appraisal. Limited knowledge of statistics and heavy clinical workload were reported as barriers to participation in a JC. Strategies such as mentoring, strong support from managers, and providing CPD (continuing professional development) points can potentially address these barriers. Opportunities for refinement of the current *i*CAHE model were raised.

**Conclusions:**

This study suggests that a structured model of JC such as *i*CAHE's model is acceptable, and likely to be used with enthusiasm by AHP to achieve EBP. Future research should explore the impact of *i*CAHE JC compared with no JC exposure, and other forms of exposure to JCs, in influencing change in allied health practitioners behaviours and evidence implementation.

## Background

This paper presents the findings of a qualitative study into the relative merits of journal clubs in allied health, in particular comparing a specific model of journal club with the experiences from traditional journal club models.

### Definition

A journal club (JC) consists of a group of individuals who meet regularly to discuss research articles in current health journals [1]. It is not a new concept and JCs have been part of many health care settings for more than a century [1,2]. However the focus of JCs has shifted over the years. It started as a way of sharing scant educational resources and a forum for discussing medical literature and keeping abreast with new knowledge [1,3]. Later on, JCs became part of most postgraduate medical education. They have been used to teach critical appraisal, research designs, and biostatistics and to improve reading habits [4-6]. In recent times, in addition to being a medium for sharing and discussing clinical cases, JCs have been considered vehicles for evidence dissemination. They have been seen as a mechanism for overcoming barriers associated with evidence-based practice [7,8].

### Application

Journal clubs have been reported in a range of health care settings, and are mostly reported for the medical and nursing professions [1,6,9]. In medicine, the use of JCs has been reported in different specialities (e.g. obstetrics and gynecology, general surgery, medicine) and different outcomes have been described. The literature reports significant improvements not only in participants' reading habits [10] but also in their knowledge of biostatistics, research design and critical appraisal [10-12]. In nursing, on the other hand, much of the literature about JCs consists of opinion papers that describe the potential benefits of JCs in promoting EBP, improving critical appraisal skills and promoting social networking among staff [13-15].

### Traditional model of journal club

In traditional models of JCs, the presenter chooses an article at random as there are no clear learning objectives for the JC [16]. The JC meeting generally consists of summarizing the article in terms of the authors' results and conclusions. Most presenters do not appraise the quality of studies, which is an important omission as a large proportion of published literature can be of poor quality [16]. The articles discussed are not necessarily relevant to clinical practice hence there is often constrained enthusiasm for ongoing attendance. Following the JC session, the article and associated information are generally not reflected upon, and any learnings are seldom processed for use in clinical practice [16].

### An innovative model of journal club -iCAHE journal club

**Figure 1:**
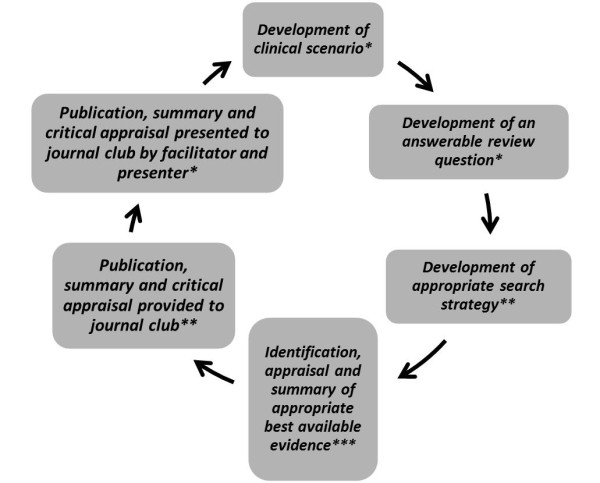
***i*CAHE journal club model**. * Tasks for the journal club; ** Tasks for the iCAHE researchers; ***Shared responsibility.

In 2007, the International Centre for Allied Health Evidence (*i*CAHE) in collaboration with the Department of Health, South Australia (SA) commenced the organisation of structured JCs across selected metropolitan and country allied health-care sites in SA. The theoretical underpinnings of the *i*CAHE JC model are based on the principles of Adult Learning or Andragogical Theory [17,18]. The *i*CAHE JC aims to provide a sustainable way of keeping allied health practitioners (AHP) informed of the current best evidence, and to ultimately promote EBP in terms of clinicians' understanding of the theory, and the application of EBP into clinical practice. Figure 1 outlines the processes involved in the model. Integral to the *i*CAHE JC is the nomination of two facilitators who will act as the point of contact between researchers at *i*CAHE and AHP at the individual site. The facilitators are required to attend a once-off training session by *i*CAHE in aspects of EBP (formulating clinical questions, searching for evidence, appraisal of evidence, implementation and evaluation of practice). Their role is to lead each JC meeting and assist members in understanding aspects of research relevant to the evidence being discussed.

The *i*CAHE model utilises a collaborative approach, where researchers and AHPs from JCs share responsibilities, as defined in Figure 1. This unique model addresses key barriers of access to, and evaluation of research evidence. It also ensures that the tasks of searching, identifying and appraising relevant literature, which have all been reported as barriers to engaging in EBP, are addressed by the involvement of researchers. In addition, participation in an *i*CAHE JC provides a supportive environment where AHP can increase their knowledge of research methodologies, share experiences and discuss current practices with colleagues, whilst focusing on translating research evidence into their usual clinical settings. Therefore, we believe that the *i*CAHE model of JC not only serves as a medium to educate AHP with the key processes involved in EBP, but it also potentially assists clinicians to address barriers associated with implementing evidence into practice.

Table 1 provides a summary of the differences that we believe are found between the traditional model of JC, and the model developed by *i*CAHE.

**Table 1:** Comparison of traditional model and *i*CAHE model of journal club

Components	Traditional model	*i*CAHE model
*Structure*	Lack of structure	Structured

*Selection of article*	Presenter chooses topic and article at random	JC chooses a topic based on current clinical problem Systematic searching of relevant articles

*Critical appraisal of the article*	Not always	Always part of JC discussion

*Support from research experts/mentor*	May seek support from a knowledgeable mentor	*i*CAHE provides training to the facilitator; provides support to the JC in aspects of searching and retrieval of literature and critical appraisal

The findings from a recent case study which evaluated the *i*CAHE JCs demonstrated that this model has the potential to address the barriers associated with searching, retrieval and critically appraising evidence from research [19]. This preliminary study suggested that the *i*CAHE JC model is helpful for clinicians to understand how to find and read literature, although it also highlighted the need to further explore its impact on learning outcomes and implementation of evidence to practice.

### Reported problems

Irrespective of which model of JC is in place, there are issues which potentially threaten the viability of the exercise, which require further exploration. These include on-going participation, compliance with attendance, sustained enthusiasm for the process and the impact of barriers to implementation of evidence identified in JCs [20,21].

The primary aim of this study is to explore the views and perspectives of AHPs regarding the use of any type of JC in promoting EBP and evidence uptake in the workplace. The secondary aim is to identify ways in which the current *i*CAHE model of JC might be improved.

## Methods

### Ethics

This study was approved by the University of South Australia Human Research Ethics Committee and the Human Research Ethics Committee (Tasmania) Network.

### Research design

A qualitative descriptive study was conducted. This is a method of naturalistic inquiry that uses low inference interpretation to present facts using everyday language [22,23]. In a qualitative descriptive study, the final product is a description of informants' views and experiences in a language similar to their own language [22,24]. In terms of analysis, therefore, we report a straight description (close to the data as recorded) of the participants' views and perspectives rather than a thick description, theory development or interpretative meaning of their experiences.

### Participants

**Figure 2:**
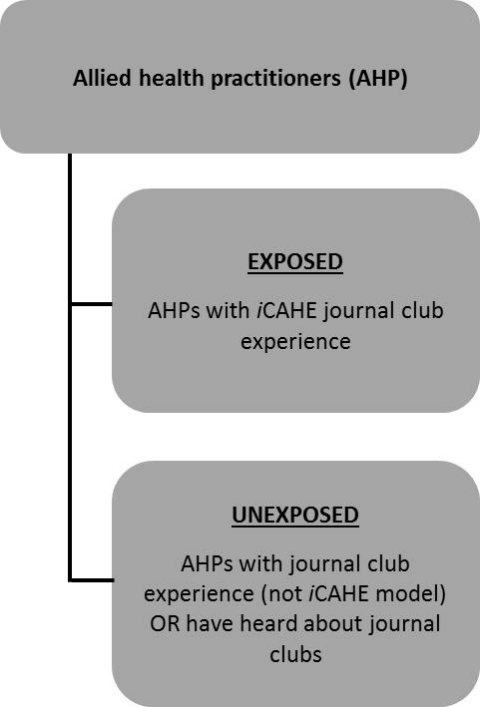
**Categories of participants**.

We included participants from two categories of AHPs (as shown in Figure 2) - those who have been exposed to the *i*CAHE JC model and those who have no experience of the iCAHE model (although they may have had exposure to other forms of JCs). Maximum variation (heterogeneity) sampling was used for two reasons. First, this strategy can capture major variations in different allied health disciplines and second, it can demonstrate shared patterns that are stable despite the variation [25].

#### Exposed participants

Over the last three years, *i*CAHE, in collaboration with the Department of Health SA, has established 30 face-to-face JCs mostly in one Australian state (South Australia). Currently, there are over 250 participants in JCs, comprising clinicians from a range of allied health disciplines. Participants for the focus group interviews were recruited by email invitation through the facilitator of the JC who is in monthly contact with a researcher from *i*CAHE. The facilitator was requested to assist with identifying knowledgeable participants for the focus group interviews. In keeping with the maximum variation sampling, we aimed to involve practitioners from every discipline, with different durations of exposure to an *i*CAHE JC, length of professional experience, educational backgrounds, roles and exposure to other EBP initiatives.

#### Unexposed participants

We invited AHPs (i.e. physiotherapists, occupational therapists, speech pathologists, social workers, psychologists, nutritionists and dieticians, and podiatrists) [26] from different health care sites to participate in the study, if they had never been exposed to an *i*CAHE JC. To avoid sample contamination, recruitment was undertaken in a city in another Australian state (Hobart, Tasmania) where iCAHE had not established any JCs. Allied health managers from different health care sites were approached to assist with identifying key informants for the focus groups. In order to maximise variation in the participants, our sampling aimed to involve practitioners from every mentioned allied health discipline, reflecting different duration of professional experience, educational background, roles (e.g. senior staff or junior staff), and exposure to research or other EBP training.

#### Organisation

The principal investigator coordinated meetings between the investigators and the facilitators of iCAHE JCs in SA/allied health managers from Tasmania. Participant information sheets describing the study were distributed to staff members during their meetings. Interested individuals were encouraged to contact the principal investigator.

### Data collection

Focus groups were conducted by the principal investigator (LL) and a co-investigator (KGS), over a one-month period. Focus groups are particularly useful when a study aims to explore participants' perspectives, by capitalising on the interaction between and among participants to stimulate and refine thoughts and perspectives [27,28]. They provide the opportunity to derive a collective perspective and validate ideas and concepts, and thus were the appropriate medium to address the study aims.

Groups consisted of six to 12 participants, with three focus groups each for the exposed and unexposed AHP. The literature suggests that this number of focus groups is usually sufficient to facilitate emergence of patterns and themes between and across groups [29,30]. For this study, focus groups were conducted until the data reached a point of saturation, (i.e. when additional information no longer generates new understanding) [27,28].

The focus group questions were semi-structured and broad. We used probes to follow up on responses and promote discussions among the participants.

Questions for the exposed group were:

1. What are your perceptions regarding the journal club that was organised in your department to promote evidence-based practice?

2. What are your impressions of how well the staff embraced the journal club project to promote evidence-based practice in your work place?

3. What are your perceptions of what works well and what does not work well within the journal club?

4. What difference did journal club participation make in your practice?

5. How do you think the journal club can be improved to better achieve its purpose?

Questions for the unexposed group were:

1. What is your understanding of evidence-based practice and journal clubs?

2. What are your views and expectations regarding the use of journal clubs as a vehicle for promoting evidence-based practice?

3. Have you been or are you currently involved with journal clubs? What are/were your experiences?

4. What factors are likely to influence the use of journal clubs by AHPs?

All focus group interviews were audio-taped, and field notes were also taken. The investigators debriefed immediately after each focus group.

### Data analysis

Audio-tapes were transcribed by an independent company. Two investigators analysed the transcribed data: the principal investigator (LL) and an experienced qualitative researcher (co-investigator (SK)) who did not participate in data collection. Using content analysis (Hancock 2002), the data were independently coded and distilled into content-related categories. The investigators compared codes and categories until consensus was reached. The categories were summarised and then confirmed with the focus group co-facilitator (KGS) to triangulate the findings. Modifications were discussed and final categories were identified.

### Investigator perspective

Two investigators (KGS, SK) were responsible for the development and organisation of *i*CAHE JCs in South Australia. The primary investigator (LL) has been the project officer of all *i*CAHE JCs.

## Results

A total of 39 (16 exposed and 23 unexposed) AHPs participated in the focus groups.

### Unexposed groups

The unexposed groups comprised 13 occupational therapists, two physiotherapists, five speech pathologists, one dietician, one social worker and one psychologist. All of them had previous exposure to a form of JC (but not the *i*CAHE JC model), either as direct participants or by knowing about one that was being conducted. Their work experience varied from a year to more than 20 years of experience. Most of the participants had a bachelor's degree, with very few having completed post graduate degrees (master's or PhD). The majority have senior position roles and few of the participants had formal training on EBP or research.

### Exposed groups

The exposed groups consisted of eight physiotherapists, four occupational therapists, two podiatrists, one dietician and one speech pathologist. Their exposure to *i*CAHE JC varied from one session to more than 25 sessions. Work experience ranged from recent graduates to over 20 years of experience. The majority had completed bachelor's degree, while a few have either completed or are in the process of finishing their master's degree. There was almost an equal distribution of participants in junior and senior positions. Less than half of the AHP in this group had had exposure to research or EBP training prior to participating in a JC.

The perspectives of unexposed and exposed AHPs on JCs were classified into five broad categories: utility and benefits of a JC, elements of an effective and sustainable JC, barriers to participation, incentives for participation, and opportunities for improvement in the current *i*CAHE JC model.

#### Utility and benefits of a journal club

The participants in both groups agreed that a JC can serve as a venue for reflective practice and keeping-up-to-date with research evidence. They all expressed that health practices should be evidence-based, and therefore AHPs should be constantly informed of the current evidence from research to inform their decisions. They also believed in the value of reflective practice, which was described as an essential attribute of health care professionals. Participants felt that being involved in a JC created an opportunity for AHP to get together and discuss their clinical practice. It provided open clinical discussion which they thought would benefit their clients.

[Unexposed] *Journal club is really useful...being able to talk about what you are doing in really simple terms and deconstruct it so you can explain it to other people. Staying up-to-date, I think that is the most useful thing. I guess the good thing about it is that, as clinicians, we don't necessarily have a lot of time to sit down and look through the literature and so if something like a journal club presents on a certain topic, it keeps me up-to-date with what's happening in that area, and how I can apply it to my practice*.

[Exposed] *Journal clubs created time for colleagues to get together and discuss research articles. We have good open clinical discussion that can only benefit the clients that we have*.

The exposed participants additionally noted that the research evidence discussed during JC meetings is valuable when making decisions about the implementation of new intervention programs for clients. Some of them highlighted how in some of their meetings, the evidence discussed validates their clinical decisions and actions.

[Exposed] *So what we've done is when we are looking to implement new programs, we actually try to find journals that report the types of programs that we're trying to run and find evidence to know if those programs are going to be beneficial*.

[Exposed] *I think the other thing is it confirms that what you are doing is in fact current and based on EBP approaches. So while you might not change things, you do realise that you're actually doing the right thing*.

The ability to appraise or critically examine the research evidence was highly regarded by participants from both groups. They stated that the knowledge and skills learnt in appraising the research articles allowed them to effectively obtain relevant information which can be integrated with their clinical decisions.

[Unexposed] *Learning to review the literature and looking at whether that information then could be used clinically within the workplace.'*

[Exposed] *This is actually useful for me...familiarising myself with how to critically read an article is very helpful. The discussion makes me understand what we need to look at, or not look at, to help make decisions*.

#### Elements of an effective and sustainable journal club

The most telling comments about effective and sustainable JCs came from the unexposed group, who had variable experiences with JCs and who had clear ideas of what they wanted if they engaged in JCs in the future. For instance, the participants in the unexposed groups identified a number of elements that they considered critical to the success of JCs. They would find it helpful to have a leader who is committed to the principles of EBP, and has a sound understanding of the processes involved. They felt that a JC leader should have knowledge of basic statistics and research and the skills to present the paper under discussion so as to appropriately and efficiently direct and assist the group achieve its goals.

[Unexposed] *To get something out of it, there should be the element of professional leadership and commitment to professional learning. Also, analysing a journal article can be difficult at times, and so a leader who has a good understanding of the issue at hand and how research evidence can be used to inform practice would be good...things like research designs, basic statistics, and critical appraisal*.

The unexposed participants expressed concern about being able to set a common time to meet, and often finding a date and time can be the most difficult part of the whole JC process. They figured it would be ideal to have a set time, which should be agreed upon by the group.

[Unexposed] *I think once a month meeting or even once every two months would be reasonable. What I thought would be effective is to set dates at the start of each year and decide as a group*.

The unexposed practitioners recognised the importance of having a clear and structured JC process to maximise the outcomes of a JC. This view stemmed from observations that many of the traditional JCs discuss randomly identified research articles which are not necessarily relevant to the members' clinical practice. As a result, the discussion becomes dominated by a few people and at times, the JC turns out to be a forum for presentation skills.

[Unexposed] *I had been involved with a journal club...would just be a rotation of people taking and discussing articles they find interesting. It wasn't always useful because people are not interested, and it was more of a show and tell rather than actually analysing the evidence*.

[Unexposed] *For me, an effective way of capturing evidence is to start with a relevant clinical question. It means a lot to me if the research article being discussed is applicable to my practice...spending time together to analyse the evidence and pull it together to come up with a conclusion and recommendations would be ideal. Discussing evidence and how it specifically relates to what we do, I think that is good. So taking that step further and making sure we always relate to it is important in a journal club structure*.

In contrast, participants in *i*CAHE JCs (Exposed group) believed that for JCs to be sustainable, there should be an on-going commitment between academics/researchers and clinicians. For many participants, there was an expectation that academics or researchers from *i*CAHE will assume a teaching role while clinicians lead the JCs. They believed that this partnership results in a blending of expertise, with *i*CAHE having the research knowledge and skills while clinicians have a better understanding of the work environment and clinical context. This raises additional issues of sustainability.

[Exposed] *I guessed the main difference I've noticed with the journal club sessions under iCAHE is that the article has been reviewed by them, which adds an extra level to the discussion because we respect the expertise that they can give*.

Times to search for and access the literature were common problems in allied health practice. Many participants reported that working together with *i*CAHE is an effective strategy to address these issues of searching and accessing EBP resources.

[Exposed] *I think for a lot of us we're reasonably time-poor on doing a lot of that research, and it's really very helpful that some of that background work has been done for us. The other good thing is, we are given access to the full text copy of the article, which in the past has always been a problem for us*.

#### Barriers to participation in a journal club

While all participants in both groups valued JCs as a medium for learning, they recognised their limitations, in that there is a distinct possibility of poor attendance and lack of active participation. Heavy clinical workload/time constraints were the most notable barriers identified by participants.

[Unexposed] *We characteristically like to do things really well and having the space and time to sit together in a journal club is hard*.

[Exposed] *I think people participate when they can but that links back to busy schedules, busy workloads, and we've just got different priorities which is always going to be a problem. Some of the times that we've chosen have clashed with some regular things that some of the allied disciplines have to do*.

Some practitioners from both groups reported that their lack of knowledge in statistics discouraged them from participating in JC discussions. They often found it difficult to understand quantitative results from journal articles. Other participants indicated that they have lost interest in JCs because they were asked to read articles which report complex statistical tests.

[Unexposed] *Some of the articles we've read have been full of statistical information, and being a non-statistician myself, it can be very difficult to get through, and to make sense of it*.

[Exposed] *I did my stats 30 years ago. I really don't have a great deal of interest in it quite frankly, so sort of knowing all the latest whizz bang tests they're talking about and all the terms sometimes is a bit beyond me*.

Another important barrier raised by practitioners from the unexposed groups is their lack of skills in searching for relevant research evidence. There were some who identified limited access to full text articles as another issue of concern. Some practitioners reported that while they are skilled in searching, limited access to electronic databases further limits them in searching and retrieving research evidence that can be useful to their practices.

[Unexposed] *I think one of the biggest burdens is not actually reading the articles, it's finding them. Sometimes your search skills aren't as crash hot as they should be, and so I end up personally just grabbing a couple of things*.

[Unexposed] Another participant added, '*Probably the biggest barrier was people getting articles, it was working for a service that didn't have the access to libraries like the department does.'*

#### Incentives for participation in a journal club

Participants in both groups described that sharing the responsibility of analysing and interpreting research evidence within the group as the leading enabler for participating in a JC meeting. They valued each other's expertise and recognised that peer support is reciprocal. Peer support offered them an opportunity not only to share their experiences but also increase their knowledge on certain areas of practice.

[Unexposed] *If you've got a group then you are more likely to ask critical questions and get the most out of it*.

[Exposed] *I think if it's spread across a lot of people it will be every single person's responsibility to do a chunk of work which will mean everyone feels a little bit more wanting to participate because it won't be as much of a demand on one person*.

The exposed and unexposed practitioners believed that what could potentially be the best incentive for increasing participation is allocation of continuing professional development (CPD) points for JC meetings. CPD or education is important in order for health practitioners to maintain, improve or broaden their knowledge and skills required in their practice. In Australia, CPD points must be earned by AHPs in order for them to renew their practicing certificate. The professional organisations allocate points, according to set criteria, to programs or activities aimed at professional development. The required CPD points vary depending on the discipline of the allied health practitioner.

[Unexposed] *We have to recruit hours for our professional registration so that's a pretty good motivator for a lot of people; it's a set way of accruing some hours towards your requirements for registration*.

[Exposed] *Look, the CPD is definitely going to change things quite significantly. Maybe if the journal clubs actually looked at contacting the actual associations so they can set it as a way of accruing some hours towards registration requirements, that's definitely a big one*.

#### Opportunities for improvement in the iCAHE journal club (Exposed groups)

Participants in *i*CAHE JC were positive about the use of JCs and expressed satisfaction in the current model. However, they believed that there were opportunities for improvements which can further increase the effectiveness and sustainability of *i*CAHE JC. Suggested refinements included training all JC members, providing self-help kits on statistics and having more regular contact with *i*CAHE researchers.

Participants stated that JC members have varied experiences and skills relevant to EBP. As such, providing training not only to facilitators but also the members can put them at the same level of understanding and thinking about EBP. They believed that, if right from the start, all the JC members have the skills to search, appraise and analyse the research evidence, JC discussions can focus on understanding the relevance and applicability of the research findings to their clinical practice.

*I would find it very helpful if the centre (iCAHE) can provide us with that education that they provide facilitators. I think that would make it so much easier for us during journal club meetings*.

Participants perceived that they now had the ability to critically appraise the literature and critically think about its significance in their practice. However, occasionally, they still experienced difficulties understanding statistics, and determining whether or not the statistical tests used are appropriate. Participants suggested that providing the JC members, as necessary, with self-help kits or 'easy-to-understand' resources about statistics can improve their level of understanding.

*I actually think a sheet with basic statistical tests, you know the more common ones, and what they actually mean, a little bit of a description of the test...sort of like a mini-statistics course I suppose would be very useful*.

For a JC to be effective and sustainable, participants felt that regular contact with 'experts' from *i*CAHE is important. They defined regular contact as having a researcher from iCAHE attend and assist in facilitating the JC at regular intervals (e.g. once every three or four meetings). Participants felt that one-day EBP training for facilitators does not make them expert facilitators.

*It's great to have a training but I also think probably having someone from your service come to one of our journal clubs and lead it, not every journal club, would be extremely helpful...particularly during the first few meetings*.

#### Summary of findings

Table 2 summarises the study findings. Overall, the exposed and unexposed groups agreed in what they perceived as benefits from JC participation, and shared similar views in how participation can be improved. Both groups recognised time constraints due to heavy clinical workload, and limited knowledge of statistics as barriers to JC involvement. While there were common findings in the way AHPs viewed JCs, there were also important differences in perspectives between practitioners from *i*CAHE JCs (exposed) and other forms of JCs (unexposed). The most notable of these differences were related to the elements believed to improve the effectiveness and sustainability of, and barriers to participation in, a JC. The exposed groups identified partnership between *i*CAHE and JCs as a critical element, while the unexposed practitioners reported good leadership, set times for meetings and a structured format for the JC as important for achieving effective and sustainable JCs. Lack of skills in searching for, and access to, the literature were barriers reported by the unexposed groups but not the exposed groups.

**Table 2:** Comparison of exposed and unexposed groups

Category	Exposed (AHP exposed to *i*CAHE JC)	Unexposed (AHP who have no experience of *i*CAHE JC, but may have had exposure to other forms of JC)
*Utility & benefits of a JC*	• Venue for reflective practice and keeping up-to-date with research evidence • Forum for learning critical appraisal of the literature	• Venue for reflective practice and keeping up-to-date with research evidence • Forum for learning critical appraisal of the literature

*Elements of an effective and sustainable JC*	• Partnership between *i*CAHE and JCs, which can address issues related to lack of time to search, and access to the literature	• Good leadership • Set time to meet • Structured format for JC

*Barriers to participation in a JC*	• Heavy clinical workload • Limited knowledge of statistics	• Heavy clinical workload • Limited knowledge of statistics • Lack of skills in searching for relevant literature • Limited access to evidence-based databases

*Incentives for participation in a JC*	• Shared responsibility within the group • Allocation of CPD points	• Shared responsibility within the group • Allocation of CPD points

*Opportunities for improvement*	• Training to all JC members • Provision of self-help kits on statistics • More regular contact with *i*CAHE researchers	--------

## Discussion

This study reports rare information on allied health JC sustainability and critical success factors, and allowed comparisons between one facilitated model of JC (*i*CAHE) and other types of JC.

Overall, practitioners believed that any JC experience was a viable tool to promote EBP uptake in allied health. Journal clubs were perceived as a vehicle for reflective practice and keeping up-to-date with the literature, and a tool for learning the critical appraisal process. The popularity of the JC in allied health can be attributed to the perceived benefits and usefulness in terms of providing a supportive environment where practitioners can learn and discuss current literature and clinical practice. This finding supports the notion that an acceptable innovation such as an evidence-based JC is one that appropriately satisfies the requirements of its users for utility and usability. Practitioners found it useful to participate in JCs that are driven by a question relevant to their day-to-day practice, and which focuses on the appropriateness of applying the findings from critically appraised evidence.

Identified elements of an effective and sustainable JC varied between those exposed to *i*CAHE JCs and other models of JCs. The elements reported by the unexposed groups exemplify the important components of the *i*CAHE JC model, which include a structured process, trained facilitators (leaders), and a set time for JC meetings. The exposed groups, on the other hand, identified partnership between *i*CAHE and JCs as a critical element. This collaboration is a unique feature of the *i*CAHE model and is intended to address issues associated with searching and access to the literature. Thus, it is not surprising that AHPs who participated in other models of JCs still reported lack of skills in searching and access to the literature as major barriers to participation. These findings validate the appropriateness of the current structure and format of the *i*CAHE JC model as a vehicle for promoting EBP uptake in allied health.

Although AHPs acknowledged the benefits of becoming involved in a JC, they cited heavy workloads and lack of skills in statistics as hindering JC participation, irrespective of the JC model in which they were engaged. Heavy workloads need to be addressed by strong administrative support for the importance of regular attendance at JC meetings. Previous studies have shown that mandatory attendance in professional development meetings may improve health practitioners' knowledge and behaviour [31,32]. On the other hand, strategies such as mentoring or training in EBP can improve practitioners' knowledge and skills in statistics [33-35].

Incentives are the factors and/or conditions that enable or encourage people to demonstrate a particular behaviour (e.g. participation in a JC). Their use in health services to promote long term sustainability of an intervention or program shows promise, but they have not been fully studied [36,37]. The participants in this study highlighted the potential impact of providing CPD points to JC participants as an incentive. CPD is an essential part of being a healthcare provider for most health professionals in Australia. Therefore, earning CPD points through JC attendance may encourage AHPs to be more actively involved in this initiative.

Regarding the innovative model of JC to which half the focus group participants had been exposed (*i*CAHE), there were suggestions to improve it, and these included providing education to all JC members, self-help kits on statistics and more regular contact with *i*CAHE researchers. In the future, *i*CAHE will endeavour to make the EBP training available to all *i*CAHE JC members as education is a prerequisite to adopting an evidence-based approach to clinical practice [38-40]. While this training includes basis statistics, it was felt that further knowledge on more complex statistics is required. Therefore, *i*CAHE will provide easy-to-understand notes on statistics relevant to the article being discussed in a JC meeting. Attendance of an *i*CAHE researcher in a JC meeting can also facilitate better understanding of research findings.

### Implications for practice

In order to achieve the best outcomes, JCs should contain the key ingredients or the critical success factors, address the identified barriers and make use of the incentive proposed by the practitioners. Based on this study, we propose the *i*CAHE JC with additional elements. These include training for all the JC members, regular contact with *i*CAHE researchers, and provision of self-help kits to improve understanding of statistics.

To present the model of JC club that we propose, we adapted a logic model developed by Harris et al [41]. Table 3 summarises the *i*CAHE model and all the important components, along with the parameters required for evaluating the outcomes of a JC and its impact on health service delivery.

**Table 3:** *i*CAHE journal club

Design	Training	Delivery	Evaluation (Outcomes) (Short term)	Evaluation (Impact) (Longer term)
• *Length of meeting *- 1hour, or dependent on the JC • *Frequency of meeting *- once every month or every other month • *Schedule of meeting - *set time agreed by the group • *Type of participants *- single discipline of allied health or multidisciplinary • *Attendance *- can be mandatory or voluntary, as set by the JC • *Size of group *- no optimal number identified	• All members - EBP principles, and processes such as formulation of clinical question, searching for evidence, appraisal of the literature • Facilitator (leader) - same training as members, but will include practical tips for running a JC • Experiential learning on how to implement evidence into practice, gained through JC discussions • Regular monitoring of JCs by *i*CAHE researchers	• Structured process (see figure 1) • Based on Adult Learning Principles • Well-defined objectives set at the start of JC • Every discussion ends with the resolution of a clinical problem and with a view towards utilising the best available evidence in making clinical decisions and evaluating its effect on practice and health care outcomes • Papers for discussion are circulated prior to the JC meeting; Critical appraisal undertaken using a structured tool	• Ability to recognise uncertainty [41] • Skills to find relevant evidence [41] • Knowledge of critical appraisal concepts, procedures [41] • Skills in summarizing the evidence [41] • Skills in using the evidence to solve problems [41]	• Willingness to apply EBP skills acquired in the journal club in the workplace [41] • Ability to use evidence to facilitate decision making [41] • Using evidence from JC to change organisation/delivery of care [41]

*[41] Harris et al (2011)

### Implications for research

Future research should explore the impact of *i*CAHE JC compared with no JC exposure, and with other forms of exposure to JCs, in influencing change in allied health practice behaviours and evidence implementation. Mixed methods research, where controlled trials are combined with qualitative approaches, should also be used to examine the outcomes of different JC styles on the various allied health disciplines, as different AHPs may have different learning styles. Further research is also needed to determine if there are other approaches that may be integrated with a JC to tailor the club according to AHPs' needs.

### Limitations

This study reported on the perceptions of AHPs as a whole. While participants had varied professional backgrounds which enhanced the representativeness of the sample, it did not provide specific information about the different disciplines. It is important to recognise that there are differences within allied health disciplines and these are widely reported in the literature [42-44]. In addition, facilitation of focus groups by the investigator who is also the project officer of *i*CAHE JC may have prohibited some participants from being critical of the *i*CAHE model. We do not believe that this is a concern however, as participants raised suggestions on how the *i*CAHE JC model can be improved.

## Conclusions

The findings indicated that AHPs were positive about the use of JCs in any form as a medium for promoting EBP. Journal clubs were perceived as a forum for reflective practice and keeping up-to-date with research evidence, and as a venue for learning the processes involved in critical appraisal. Clinical workload and limited knowledge of statistics were reported as major barriers to participation in JC meetings. Strategies such as mentoring or training, and strong support from managers, peers and researchers/academics can potentially address these barriers. This study suggests that a structured model of journal club such as *i*CAHE's model is acceptable, and likely to be used with enthusiasm by AHPs to achieve EBP. Opportunities for refinement of the current *i*CAHE model were raised.

## Competing interests

The authors declare that they have no competing interests.

## Authors' contributions

LML conceived of the study, collected and analysed data, and drafted the manuscript. KGS collected and analysed data and helped draft the manuscript. SK participated in the design of the study, analysed data and helped draft the manuscript. All authors read and approved the final manuscript.

## Pre-publication history

The pre-publication history for this paper can be accessed here:

http://www.biomedcentral.com/1472-6920/11/66/prepub
